# Resting Cardiac Power Predicts Adverse Outcome in Heart Failure Patients With Preserved Ejection Fraction: A Prospective Study

**DOI:** 10.3389/fcvm.2022.915918

**Published:** 2022-07-05

**Authors:** Shiqi Wang, Aiqi Chen, Xiaokai Duan

**Affiliations:** ^1^General Department of Zhengzhou First People’s Hospital, Zhengzhou, China; ^2^Department of Cardiology, Hospital of Joint Logistic Support Force of the Chinese People’s Liberation Army, Zhengzhou, China

**Keywords:** cardiac power, heart failure with preserved ejection fraction, echocardiography, prognosis, adverse outcome

## Abstract

**Background:**

We sought to explore the significance of resting cardiac power/mass in predicting adverse outcome in patients with heart failure with preserved ejection fraction (HFpEF).

**Methods:**

This prospective cohort study included patients with HFpEF and without significant valve disease or right ventricular dysfunction. Cardiac power was normalized to left ventricular (LV) mass and expressed in W/100 g of LV myocardium. Multivariate Cox regression analysis was used to evaluate the association between resting cardiac power/mass and composite endpoint, which included all-cause mortality and heart failure (HF) hospitalization.

**Results:**

A total of 2,089 patients were included in this study. After an average follow-up of 4.4 years, 612 (29.30%) patients had composite endpoint, in which 331 (15.84%) died and 391 (18.72%) experienced HF hospitalization. In multivariate Cox regression analysis, resting power/mass < 0.7 W/m^2^ was independently associated with composite endpoint, all-cause mortality, cardiovascular mortality and HF hospitalization, with hazard ratios (HR) of 1.309 [95% confidence interval (CI): 1.108–1.546, *P* = 0.002], 1.697 (95%CI: 1.344–2.143, *P* < 0.001), 2.513 (95%CI: 1.711–3.689, *P* < 0.001), and 1.294 (95%CI: 1.052–1.592, *P* = 0.015), respectively. For composite endpoint, cardiovascular mortality and HF hospitalization, the C statistic increased significantly when incorporating resting cardiac power/mass into a model with established risk factors. For composite endpoint, the continuous net reclassification index after adding resting cardiac power/mass in the original model with N-terminal pro-brain natriuretic peptide was 13.1% (95%CI: 2.9–21.6%, *P* = 0.007), and the integrated discrimination index was 1.9% (95%CI: 0.8–3.2%, *P* < 0.001).

**Conclusion:**

Resting cardiac power determined by non-invasive echocardiography is independently associated with the risk of adverse outcomes in HFpEF patients and provides incremental prognostic information.

## Introduction

Chronic heart failure (HF), characterized by decreased cardiac systolic and/or diastolic function, is a primary cause of morbidity and mortality worldwide (1). A primary concern in HF management lies in accurately and comprehensively evaluating cardiac function, especially left ventricular (LV) function, as the left ventricle is responsible for most of the physiological functions of the heart. When evaluating LV function, clinicians usually adopt the traditional indexes including LV ejection fraction (EF) and cardiac output. Although well known, these measurements may fail to provide a comprehensive assessment of LV function. In addition, nearly half of all HF patients have preserved EF (namely HFpEF) (2). Thus, EF alone seems inadequate to identify high-risk individuals among HF patients.

Cardiac power refers to the rate at which the heart pumps blood out and delivers it to the periphery (3). Thus, it is an integrative and quantitative indicator of overall cardiac performance that combines cardiac pressure and volume loads. Normally, 3 mmHg of right atrial pressure, 120/80 mmHg of arterial pressure, and 5 L/min of cardiac output produce ∼1 W of resting cardiac power. Some studies have shown that higher resting and peak cardiac power are associated with a lower mortality in patients with chronic and advanced HF (4, 5). Furthermore, a retrospective study conducted by Anand et al. (6) showed that in patients with normal EF and no HF undergoing exercise stress echocardiography, higher cardiac power was independently associated with a lower prevalence of complications, as well as a lower risk of all-cause mortality and the incidence of subsequent HF, suggesting the potential prognostic value of cardiac power.

Whereas cardiac power has been well studied in HF patients with reduced EF (HFrEF) and in those with normal EF but no HF (4–7), its significance and role in predicting adverse outcomes in patients with HFpEF remains unclear. Thus, in this study we aimed to evaluate the role of cardiac power indexed to LV mass in patients with stable HFpEF.

## Materials and Methods

This study was approved by the Ethics Board of the No. 988 Hospital of Joint Logistic Support Force of the Chinese PLA and was conducted in line with the ethical guidelines of the 1975 Declaration of Helsinki. Written informed consent was obtained from each patient. The datasets used and/or analyzed during the current study are available from the corresponding author on reasonable request.

### Participants

This prospective cohort study recruited patients who were hospitalized in the Cardiology Department of the No. 988 Hospital of Joint Logistic Support Force of the Chinese PLA (Zhengzhou, China) from April 2012 to December 2021. We used the following criteria to select HFpEF patients: those with (i) history of HF hospitalization; (ii) HF syndromes and/or signs, (iii) EF ≥ 50%, and (iv) N-terminal pro-brain natriuretic peptide (NT-proBNP) ≥ 125 pg/mL. Additionally, eligible HFpEF patients were required to be stable and well-compensated without medication changes for at least 6 weeks prior to enrollment. Patients who had one of the following conditions were excluded: EF < 50% at rest, enlarged right ventricle, significant valve disease (≥ moderate stenosis or regurgitation, prosthetic valve replacement, surgical or percutaneous valve repair, rheumatic valve disease), hospitalization for uncompensated HF or unstable coronary heart disease in the prior 6 weeks, heart transplant, metastatic malignant tumor, severe liver disease or receiving palliative care. Via electronic medical records, we collected patient detailed medical history, baseline clinical characteristics, laboratory indexes and echocardiographic parameters.

### Resting Cardiac Power and Comorbidity Score

Cardiac power normalized by LV mass at rest was calculated by the following formulas, in which 0.222 is the conversion constant to W/100 g of LV myocardium: resting cardiac power/mass = 0.222 × cardiac output × mean blood pressure (BP)/LV mass; cardiac output = stroke volume × heart rate; mean BP = diastolic BP + 1/3 × systolic BP (5). In each patient, stroke volume was calculated from the product of EF (biplane modified Simpson’s method) and LV end-diastolic volume by 2D echocardiography, assuming normal LV geometry, which is a reasonable assumption in the situation of normal EF, no right ventricular dysfunction, and no significant valvular heart disease (6). The following formula was used to calculate LV mass: LV mass (g) = 0.8 × 1.04 × [(interventricular septal thickness + LV end diastolic diameter + LV posterior wall thickness)^3^ - (LV end diastolic diameter)^3^] + 0.6 (8). Indexing each measure for body surface area is not needed since their ratio takes care of this normalization.

To evaluate the severity of patient comorbidities, we defined comorbidity score as the number of patient comorbidities referring to the Charlson Comorbidity Index (CCI) (9). For the present study, we included the comorbidities that score 1 point in the CCI (myocardial infarction, peripheral vascular disease, cerebrovascular disease, dementia, chronic lung disease, connective tissue disease, ulcer disease, mild liver disease and diabetes), as well as anemia, hypertension and atrial fibrillation, and excluded those comorbidities scoring ≥ 2 points in CCI (such as severe liver disease, metastatic tumors and leukemia). As our analysis was entirely among HFpEF patients, the 1 point added for HF was not included in the final number of comorbidities.

### Follow-Up and Outcomes

Until December 31, 2021, all patients were followed up via telephone or medical record every 6 months for the composite endpoint which consisted of all-cause mortality or HF hospitalization, and the causes of death was also recorded. Furthermore, we contacted the attending physician of each patient who had an event to reconfirm their outcome. For patients who did not have an event, survival time was defined as the period from the day of physical examination to the last date of follow-up.

### Statistical Analysis

Categorical variables are presented as frequencies (%), and continuous variables are presented as the mean ± standard deviation or median (interquartile range). Differences between groups were evaluated by the chi-squared test for categorical variables and Student’s *t*-test or the Mann–Whitney *U*-test for continuous variables, as appropriate.

We log-transformed (log_10_) NT-proBNP, and used the median values of resting cardiac power/mass and log NT-proBNP as cutoffs. The log-rank test was used to compare survival times on Kaplan–Meier curves across different groups. The prognostic value of resting cardiac power/mass was evaluated by using a Cox proportional hazards model adjusted for the following covariates: age, gender, body mass index (BMI, calculated by weight divided by the square of height), New York Heart Association (NYHA) class, LVEF, comorbidity score, estimated glomerular filtration rate (eGFR, calculated by a modified Modification of Diet in Renal Disease equation), angiotensin-converting enzyme inhibitors (ACEI)/angiotensin receptor antagonists (ARB), beta blockers and aldosterone antagonists. The prognostic discrimination of resting cardiac power/mass was assessed by comparing the incremental improvement of the Harrell’s C statistic, as well as the integrated discrimination improvement (IDI) and the continuous net reclassification improvement (NRI) at the event rate. Sensitivity analysis was further performed to explore the association between resting cardiac power/mass (as a continuous variable) and all-cause mortality and HF hospitalization among the following subgroups: age (< 75 or ≥ 75 years), BMI (< 18.5 kg/m^2^, 18.5–23.9 kg/m^2^, 24–27.9 kg/m^2^ or ≥ 28 kg/m^2^), NYHA class (class I, II, III or IV), comorbidity score (0–3 or ≥ 4), eGFR (< 60 mL/min/1.73 m^2^ or ≥ 60 mL/min/1.73 m^2^) and log NT-proBNP (< 2.5 or ≥ 2.5). R software version 4.0.3 (Institute for Statistics and Mathematics, Vienna, Austria^1^) and SPSS 26.0 software (IBM Corporation, Armonk, NY, United States) were used to perform statistical analyses. A *P*-value < 0.05 was considered significant.

## Results

### Baseline Characteristics

Exclusion of ineligible patients produced a final cohort of 2089 HFpEF patients. Baseline measurements of resting cardiac power/mass were available for the 2089 patients. Detailed baseline characteristics are shown in Table 1. The median value of resting cardiac power/mass was 0.7 W/m^2^, which was used as a cutoff value for grouping. Compared with patients with resting cardiac power/mass ≥ 0.7 W/m^2^, those with resting cardiac power/mass < 0.7 W/m^2^ were older, had higher NT-proBNP, more comorbidities and larger percentage of NYHA class IV, ischemic heart disease and atrial fibrillation, used more cardiovascular medications, while had lower eGFR. We listed the echocardiography parameters of patients in Supplementary Table 1, which showed that compared with patients with resting cardiac power/mass ≥ 0.7 W/m^2^, those with resting cardiac power/mass < 0.7 W/m^2^ had larger interventricular septal thickness, left ventricular posterior wall thickness, tricuspid regurgitation velocity, left ventricular end systolic volume, left ventricular mass index, and relative wall thickness (all *P* < 0.05), besides, the former also had larger left atrial volume index than the latter, though without statistical significance (*P* = 0.068).

**TABLE 1:** Baseline characteristics of patients with HFpEF.

	Resting cardiac power/mass < 0.7 W/m^2^ (*N* = 1,021)	Resting cardiac power/mass ≥ 0.7 W/m^2^ (*N* = 1,068)	*P*
Age (years)	77.90 ± 11.37	74.78 ± 11.39	<0.001
Male (%)	983 (96.28%)	1,018 (95.32%)	0.163
Smoking (%)	376 (37.12%)	386 (36.28%)	0.363
Alcohol (%)	326 (32.06%)	339 (31.83%)	0.475
BMI (kg/m^2^)	24.48 ± 2.93	24.52 ± 3.21	0.788
Systolic BP (mmHg)	128.67 ± 16.72	137.40 ± 16.60	<0.001
Diastolic BP (mmHg)	66.66 ± 8.80	75.41 ± 9.50	<0.001
Heart rate (bpm)	66.56 ± 8.50	77.10 ± 10.49	<0.001
**NYHA class (%)**			
I	149 (14.59%)	151 (14.14%)	0.767
II	445 (43.58%)	505 (47.28%)	0.090
III	317 (31.05%)	325 (30.43%)	0.760
IV	110 (10.77%)	87 (8.15%)	0.040
eGFR (mL/min/1.73 m^2^)	87.79 ± 24.94	90.36 ± 23.52	0.016
FBG (mmol/L)	5.78 ± 1.61	5.81 ± 1.72	0.654
NT-proBNP (pg/mL)	416.9 (257.43–828.55)	345.6 (228.05–617.60)	0.001
LVEF (%)	61 (58–64)	63 (61–66)	<0.001
Comorbidity score	3.52 ± 1.90	3.15 ± 1.81	<0.001
Ischemic heart disease (%)	522 (51.13%)	412 (38.58%)	<0.001
Hypertension (%)	671 (65.72%)	706 (66.10%)	0.853
Atrial fibrillation (%)	163 (15.96%)	113 (10.58%)	<0.001
Diabetes mellitus (%)	464 (45.45%)	496 (46.44%)	0.648
**Medication (%)**			
Anti-platelet	732 (71.69%)	677 (63.39%)	<0.001
Statins	701 (68.66%)	626 (58.61%)	<0.001
Calcium channel blocker	621 (60.82%)	608 (56.93%)	0.071
ACEI/ARB	563 (55.14%)	539 (50.47%)	0.032
Beta blocker	506 (49.56%)	415 (38.86%)	<0.001
Aldosterone antagonist	276 (27.03%)	227 (21.25%)	0.002
Diuretic	549 (53.77%)	472 (44.19%)	<0.001

*HFpEF, heart failure with preserved ejection fraction; BMI, body mass index; BP, blood pressure; bpm, beats per minute; NYHA, New York Heart Association; eGFR, estimated glomerular filtration rate; FBG, fasting blood glucose; NT-proBNP, N-terminal pro-brain natriuretic peptide; LVEF, left ventricular eject fraction; ACEI, angiotensin-converting enzyme inhibitors; ARB, angiotensin receptor antagonist.*

### Clinical Outcomes

The 2089 HFpEF patients were followed up for 4.4 years on average, with no patients lost to follow-up. Among them, 612 (29.30%) patients experienced composite endpoint, in which 331 (15.84%) experienced death from any cause, 147 (7.04%) died from cardiovascular causes, and 391 (18.72%) experienced HF hospitalization. Of these, 357 (34.97%) patients who had composite endpoint, 216 (65.26%) patients who died and 225 (57.54%) patients hospitalized with HF had resting cardiac power/mass values below 0.7 W/m^2^.

**FIGURE 1:**
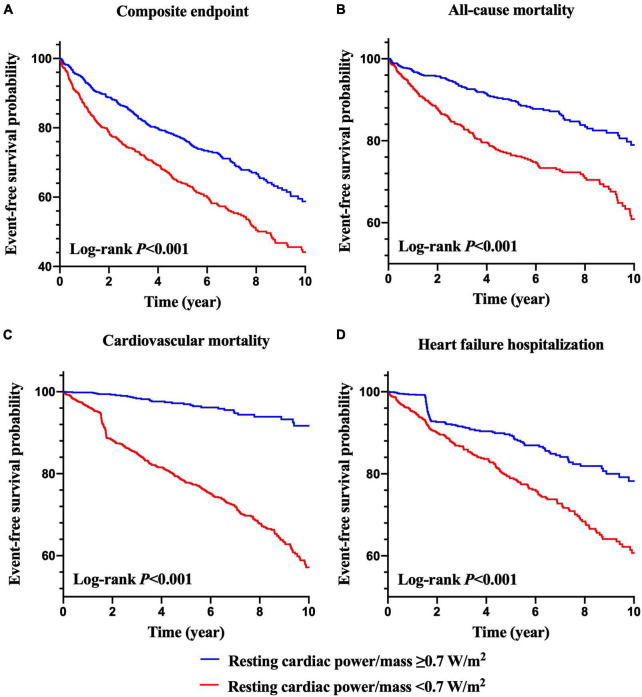
Kaplan–Meier survival curves for prediction of composite endpoint **(A)**, all-cause mortality **(B)**, cardiovascular mortality **(C)** and heart failure hospitalization **(D)** in patients with higher resting cardiac power/mass (≥ 0.7 W/m^2^) and lower resting cardiac power/mass (< 0.7 W/m^2^).

**FIGURE 2:**
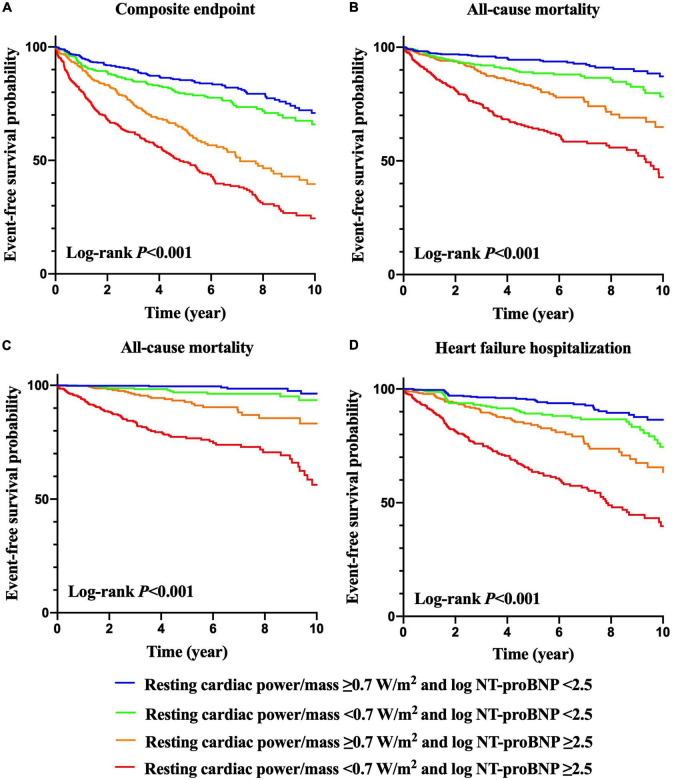
Kaplan–Meier survival curves for prediction of composite endpoint **(A)**, all-cause mortality **(B)**, cardiovascular mortality **(C)** and heart failure hospitalization **(D)** according to resting cardiac power/mass and NT-proBNP levels.

Kaplan–Meier curves of the incidences of adverse outcomes are presented in Figures 1, 2 across different category methods. The incidences of adverse outcomes in patients with resting cardiac power/mass < 0.7 W/m^2^ were significantly higher than those with resting cardiac power/mass ≥ 0.7 W/m^2^ (each log-rank *P* < 0.001, Figure 1). To compare the outcomes of patients with different levels of cardiac power/mass and NT-proBNP, we divided the cohort into four groups based upon the median values of resting cardiac power/mass and log NT-proBNP (Figure 2). Patients with either a lower resting cardiac power/mass or higher NT-proBNP had an increased risk of meeting either endpoint compared with the reference group that had higher resting cardiac power/mass and lower NT-proBNP (*P* < 0.001).

After adjustment for commonly recognized risk factors (age, gender, BMI, NYHA class, LVEF, comorbidity score, eGFR, use of ACEI/ARB, beta blocker and aldosterone antagonist, log NT-proBNP), in multivariate analysis, resting cardiac power/mass < 0.7 W/m^2^ was independently associated with the incidence of composite endpoint, all-cause mortality, cardiovascular mortality and HF hospitalization, with hazard ratios (HR) of 1.309 [95% confidence interval (CI): 1.108–1.546, *P* = 0.002], 1.697 (95%CI: 1.344–2.143, *P* < 0.001), 2.513 (95%CI: 1.711–3.689, *P* < 0.001), and 1.294 (95%CI: 1.052–1.592, *P* = 0.015), respectively (Table 2).

**TABLE 2:** Outcomes of HFpEF patients by resting cardiac power/mass categories.

Outcomes	Resting cardiac power/mass ≥ 0.7 W/m^2^ (*N* = 1,068)	Resting cardiac power/mass < 0.7 W/m^2^ (*N* = 1,021)	*P*
		
	HR (95%CI)	HR (95%CI)	
**Composite endpoint**			
*N* (%)	255 (23.88%)	357 (34.97%)	
Unadjusted	1.00 (Ref)	1.654 (1.408–1.943)	<0.001
Model	1.00 (Ref)	1.349 (1.142–1.593)	<0.001
Model + log NT-proBNP	1.00 (Ref)	1.309 (1.108–1.546)	0.002
**All-cause mortality**			
*N* (%)	115 (10.77%)	216 (21.16%)	
Unadjusted	1.00 (Ref)	2.213 (1.764–2.775)	<0.001
Model	1.00 (Ref)	1.726 (1.367–2.180)	<0.001
Model + log NT-proBNP	1.00 (Ref)	1.697 (1.344–2.143)	<0.001
**Cardiovascular mortality**			
*N* (%)	37 (3.46%)	110 (10.77%)	
Unadjusted	1.00 (Ref)	3.549 (2.445–5.151)	<0.001
Model	1.00 (Ref)	2.541 (1.727–3.737)	<0.001
Model + log NT-proBNP	1.00 (Ref)	2.513 (1.711–3.689)	<0.001
**HF hospitalization**			
*N* (%)	166 (15.54%)	225 (22.04%)	
Unadjusted	1.00 (Ref)	1.582 (1.294–1.933)	<0.001
Model	1.00 (Ref)	1.331 (1.065–1.613)	0.011
Model + log NT-proBNP	1.00 (Ref)	1.294 (1.052–1.592)	0.015

*Model is adjusted for age, gender, BMI, NYHA class, LVEF, comorbidity score, eGFR, ACEI/ARB, beta blocker and aldosterone antagonist. HR, hazard ratio; CI, confidence interval; other abbreviations are same as Table 1.*

### Incremental Value of Resting Cardiac Power/Mass for All-Cause Mortality and Heart Failure Hospitalization

We further explored the predictive value of resting cardiac power/mass by C-index (Table 3). The individual addition of log NT-proBNP or resting cardiac power/mass in the model significantly improved the C statistic for predicting composite endpoint, cardiovascular mortality, and HF hospitalization. Also, the two indicators significantly increased the C statistic for predicting adverse outcomes when they were incorporated into the established model.

**TABLE 3:** Reclassification and discrimination statistics for outcomes by resting cardiac power/mass.

Outcomes	C-index (95%CI)	*P*	Continuous NRI (%, 95%CI)	*P*	IDI (%, 95%CI)	*P*
**Composite endpoint**						
Model	0.721 (0.699–0.743)		1.0 (Ref)		1.0 (Ref)	
Model + log NT-proBNP	0.739 (0.722–0.756)	0.018	8.4 (3.0–18.0)	0.133	1.4 (0.1–2.9)	0.033
Model + resting cardiac power/mass	0.726 (0.714–0.738)	0.005	13.4 (1.8–23.2)	0.013	0.6 (0.1–1.1)	0.020
Model + log NT-proBNP + resting cardiac power/mass	0.742 (0.729–0.755)	0.021	13.1 (2.9–21.6)	0.007	1.9 (0.8–3.2)	<0.001
**All-cause mortality**						
Model	0.741 (0.712–0.770)		1.0 (Ref)		1.0 (Ref)	
Model + log NT-proBNP	0.753 (0.724–0.781)	0.011	8.0 (3.0–25.7)	0.013	1.6 (0.1–3.0)	0.040
Model + resting cardiac power/mass	0.748 (0.719–0.777)	0.060	13.8 (2.4–24.0)	0.020	0.9 (0.1–1.8)	0.004
Model + log NT-proBNP + resting cardiac power/mass	0.759 (0.731–0.788)	0.001	17.0 (11.4–28.3)	0.040	2.3 (0.7–8.7)	0.020
**Cardiovascular mortality**						
Model	0.862 (0.832–0.892)		1.0 (Ref)		1.0 (Ref)	
Model + log NT-proBNP	0.891 (0.872–0.910)	0.029	22.3 (3.7–47.4)	0.140	4.5 (1.0–9.5)	<0.001
Model + resting cardiac power/mass	0.877 (0.858–0.896)	0.014	25.2 (2.9–49.4)	0.040	2.5 (0.1–6.0)	0.047
Model + log NT-proBNP + resting cardiac power/mass	0.902 (0.873–0.931)	0.040	33.1 (4.9–55.3)	0.007	7.5 (2.2–14.4)	<0.001
**HF hospitalization**						
Model	0.717 (0.689–0.745)		1.0 (Ref)		1.0 (Ref)	
Model + log NT-proBNP	0.747 (0.721–0.773)	<0.001	4.0 (1.7–15.0)	0.027	1.3 (1.2–3.9)	0.077
Model + resting cardiac power/mass	0.723 (0.696–0.750)	0.037	4.6 (2.3–14.9)	0.006	0.5 (0.1–1.5)	0.058
Model + log NT-proBNP + resting cardiac power/mass	0.749 (0.723–0.775)	<0.001	6.0 (4.7–15.2)	0.026	1.7 (1.2–4.3)	0.007

*Model is adjusted for age, gender, BMI, NYHA class, LVEF, comorbidity score, eGFR, ACEI/ARB, beta blocker and aldosterone antagonist. CI, confidence interval; NRI, net re-classification improvement; IDI, integrated discrimination improvement; other abbreviations are same as Table 1.*

For composite endpoint, the continuous NRI after adding resting cardiac power/mass in the original model with N-terminal pro-brain natriuretic peptide was 13.1% (95%CI: 2.9–21.6%, *P* = 0.007), and the IDI was 1.9% (95%CI: 0.8–3.2%, *P* < 0.001). For all-cause mortality, the continuous NRI after the addition of resting cardiac power/mass in the model with established risk factors and NT-proBNP was 17.0% (95%CI: 11.4–28.3%, *P* = 0.040), and the IDI was 2.3% (95%CI: 0.7–8.7%, *P* = 0.020). For cardiovascular mortality, the continuous NRI after the addition of resting cardiac power/mass in the model with established risk factors and NT-proBNP was 33.1% (95%CI: 4.9–55.3%, *P* = 0.007), and the IDI was 7.5% (95%CI: 2.2–14.4%, *P* < 0.001). For HF hospitalization, the continuous NRI after the addition of resting cardiac power/mass in the model with established risk factors and NT-proBNP was 6.0% (95%CI: 4.7–15.2%, *P* = 0.026), and the IDI was 1.7% (95%CI: 1.2–4.3%, *P* = 0.007). We additionally compared the predictive value between NT-proBNP and resting cardiac power/mass, and listed the results in Supplementary Table 2, which showed that the addition of rest cardiac power/mass improved the predictive value of the model based on NT-proBNP.

### Resting Cardiac Power/Mass as an Independent Predictor of Outcomes

**FIGURE 3:**
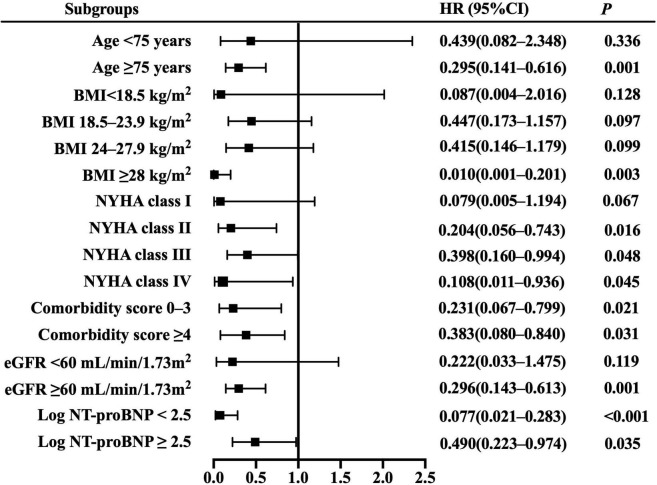
Resting cardiac power/mass for the prediction of all-cause mortality: subgroup analysis. The prognostic value of resting cardiac power/mass is considered in several patient subgroups, after adjustment for age, gender, BMI, NYHA class, LVEF, comorbidity score, eGFR, log NT-proBNP, ACEI/ARB, beta blocker, and aldosterone antagonist. Other abbreviations as in Table 1.

**FIGURE 4:**
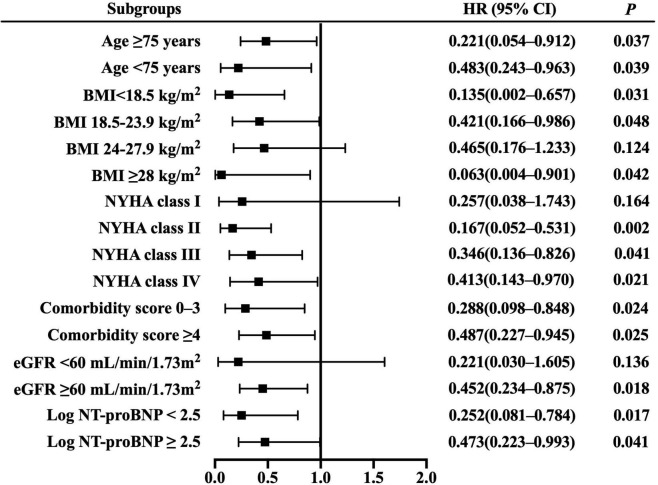
Resting cardiac power/mass for the prediction of heart failure hospitalization: subgroup analysis. The prognostic value of resting cardiac power/mass is considered in several patient subgroups, after adjustment for age, gender, BMI, NYHA class, LVEF, comorbidity score, eGFR, log NT-proBNP, ACEI/ARB, beta blocker, and aldosterone antagonist. Other abbreviations as in Table 1.

Resting cardiac power/mass was independently associated with all-cause mortality across most patient subgroups (Figure 3), including the groups of age ≥ 75 years, BMI ≥ 28 kg/m^2^, NYHA class II– IV, comorbidity score 0–3 and ≥ 4, eGFR ≥ 60 mL/min/1.73 m^2^, and log NT-proBNP < 2.5 and ≥ 2.5. Similarly, it presented independent prognostic value for HF hospitalization in multiple subsets (Figure 4), including the groups of age ≥ 75 and < 75 years, BMI < 18.5,18.5–23.9 and ≥ 28 kg/m^2^, NYHA class II– IV, comorbidity score 0–3 and ≥ 4, eGFR ≥ 60 mL/min/1.73 m^2^, and log NT-proBNP < 2.5 and ≥ 2.5. Combined with the results shown in Table 2, the sensitivity analyses indicate that resting cardiac power/mass is an independent predictor for these adverse outcomes in patients with stable HFpEF.

## Discussion

In the present study, we comprehensively investigated the potential prognostic role of cardiac power estimated by non-invasive echocardiography in patients with stable HFpEF. In this study, we found that (i) cardiac power normalized to LV mass at rest was independently associated with adverse outcomes in patients with HFpEF, and that (ii) incorporating resting cardiac power/mass (reflective of comprehensive cardiac function) and NT-proBNP (indicative of myocardial stretch) into a model with established risk factors enhanced the prognostic value for those endpoints.

As is well known, LV diastolic dysfunction plays a fundamental and predominant role in the pathophysiology of HFpEF (10) and elevates filling pressure, which further promotes dyspnea, impairs exercise capacity, and increases mortality and incidence of HF hospitalization (11). Non-invasive echocardiography is an indirect approach to measuring LV diastolic function. Due to its safety and rapidity, echocardiography is usually preferred for clinicians before performing invasive right heart catheterization. However, individual parameters of echocardiography, such as left atrial volume index and the mitral annular diastolic velocity, have their own limitations and may fail to accurately determine the degree as well as the severity of LV dysfunction. Moreover, a combination of these parameters undoubtedly increases the work burden on clinical staff and is not conducive to clinical application.

Despite having a “preserved” EF, patients with HFpEF nonetheless experience abnormalities in LV systolic performance (12, 13). Multiple studies have demonstrated that patients with HFpEF present with subtle impairments in systolic function at rest, and this alteration tends to increase during exertion, which impairs LV suction, decreases cardiac output, and elevates LV filling pressures (14, 15). LV systolic dysfunction in HFpEF also predicts increased risk of adverse outcomes (16, 17). Some scholars question the ability of EF to truly reflect the LV systolic function of HFpEF (18). HFpEF is often complicated with myocardial concentric hypertrophy, which inevitably generates a normal or supernormal EF, even when stroke volume has declined. Therefore, a non-invasive method that can quantify LV pump function better than EF would be a major step forward.

Cardiac power is a comprehensive quantitative indicator that can be used to evaluate cardiac function via non-invasive echocardiography (4, 19). It is superior to variables such as cardiac output because it covers both pressure load and volume load (20). Because cardiac power depends on the volume of the muscle that produces the power, standardizing cardiac power with LV mass can improve the applicability of this indicator in different populations (4, 5, 19) and facilitate comparison between individuals. In addition, cardiac power may be more promising than EF as predictor of mortality in severe heart failure. It has been reported to be the strongest predictor of in-hospital mortality in patients with cardiac shock resulting from acute myocardial infarction (20), and has recently been used to evaluate the response of patients with advanced HF to mechanical circulatory support systems (21).

In the present study, we found that the HFpEF patients with lower resting cardiac power/mass were more likely to be older, used more cardiovascular medications, have higher NYHA class and NT-proBNP level, as well as more comorbidities, indicating that the patients in this subgroup might have poorer health at baseline and more risk factors for adverse outcomes. After adjustment for multiple covariates, such as age and comorbidities, our study brought new evidence that resting cardiac power/mass is independently associated with composite endpoint, all-cause mortality, cardiovascular mortality, and HF hospitalization in patients with HFpEF. Meanwhile, resting cardiac power/mass significantly promoted the prediction efficiency of both traditional risk factors and NT-proBNP, supporting a pathophysiological link between reduced cardiac performance and the mortality and HF progression later in life as aging and comorbidities advance. Furthermore, the results of our sensitivity analysis showed that although not all subgroups showed a statistically significant association, the risk of adverse outcomes within these subgroups was higher in patients with lower resting cardiac power/mass than in those with higher resting cardiac power/mass, indicating the stable and independent prediction efficacy of resting cardiac power/mass among HFpEF patients. Clinically, resting cardiac power/mass is easily obtained by measuring blood pressure and stroke volume, the latter of which can be measured by Doppler echocardiography. Intriguingly, the technical setup used to determine cardiac power is very similar to that used in a standard diastolic stress test, so determining whether integrated application of the two tests may provide incremental diagnostic or prognostic significance in HFpEF patients deserves further exploration.

Our study has some limitations: first, it should be noted that this was a single-center study and 95.79% of our patients were male because we included patients from the veteran population, thus we failed to observe the association between rest cardiac power and adverse outcomes in gender subgroup, and our conclusion might be more suitable for male patients. We further conducted Cox proportional hazard model and adjusted gender, age and other covariates, and the results were still significant, suggesting that rest cardiac power/mass predicts the adverse outcomes in HFpEF patients independent of gender, therefore the results of this study were still representative to some extent, and studies conducted with more female patients are needed to validate our findings. Secondly, compared with invasive measurements, the non-invasive measurements of stroke volume may be more inclined to error. Thirdly, this study failed to perform speckle tracking echocardiography and thus lacked the information of global longitudinal strain, which helps determine the impaired systolic function in patients with normal ejection fraction and should be fully considered in the further studies. Last, the prognostic value of peak or reserved cardiac power indexed to LV mass has not been evaluated in patients with HFpEF.

## Conclusion

This study explored the association between resting cardiac power/mass and the risk of adverse outcomes in patients with HFpEF, finding that lower resting cardiac power/mass is an independent predictor of these adverse outcomes and also has incremental prognostic value over established risk factors and NT-proBNP. Cardiac power as an integrated indicator of cardiac performance may be considered for risk stratification of long-term adverse outcomes in patients with HFpEF. This measurement provides more comprehensive and accurate guidance for treatment and prognostic evaluation of patients with HFpEF, and further promotes the integration and optimization of cardiac function monitoring indicators.

## Data Availability Statement

The original contributions presented in this study are included in the article/Supplementary Material, further inquiries can be directed to the corresponding author.

## Ethics Statement

This study was approved by the Ethics Board of the No. 988 Hospital of Joint Logistic Support Force of the Chinese PLA. The patients/participants provided their written informed consent to participate in this study.

## Author Contributions

SW designed the protocol, provided methodological expertise, drafted the manuscript and performed statistical analyses. AC and XD supervised patient recruitment and study procedures and conducted study procedures. All authors read and approved the final manuscript.

## Conflict of Interest

The authors declare that the research was conducted in the absence of any commercial or financial relationships that could be construed as a potential conflict of interest.

## Publisher’s Note

All claims expressed in this article are solely those of the authors and do not necessarily represent those of their affiliated organizations, or those of the publisher, the editors and the reviewers. Any product that may be evaluated in this article, or claim that may be made by its manufacturer, is not guaranteed or endorsed by the publisher.

## Footnotes

1
http://cran.r-project.org
