# Supplementary material for: Resting Cardiac Power Predicts Adverse Outcome in Heart Failure Patients With Preserved Ejection Fraction: A Prospective Study
Source: Front Cardiovasc Med. 2022 Jul 5;9:915918. doi: 10.3389/fcvm.2022.915918 (PMC9294213; doi:10.3389/fcvm.2022.915918)
Supplement: Supplementary file 1 [file Table_1.DOCX]

Supplementary Material

Supplementary Table 1. Echocardiography parameters of patients

|  | Resting cardiac power/mass <0.7 W/m^2^ (N=1021) | Resting cardiac power/mass ≥0.7 W/m^2^ (N=1068) | *P* |
| --- | --- | --- | --- |
| IVS (mm) | 11.44±1.45 | 11.07±1.26 | <0.001 |
| LVPWT (mm) | 11.07±0.96 | 10.95±0.87 | 0.002 |
| LVEDD (mm) | 53.04±1.74 | 53.05±1.52 | 0.206 |
| LVESD (mm) | 36.10±3.34 | 36.19±2.69 | 0.201 |
| LAD (mm) | 43.67±3.35 | 43.51±3.30 | 0.261 |
| TR velocity (m/s) | 3.15±0.12 | 2.94±0.14 | 0.004 |
| LVEDV (mL) | 143.55±8.38 | 143.08±7.33 | 0.176 |
| LVESV (mL) | 56.59±8.69 | 52.95±6.69 | <0.001 |
| LVMI (g/m^2^)* | 134.60±20.72 | 129.23±17.07 | <0.001 |
| LVAI (mL/m^2^)** | 27.74±5.11 | 27.34±4.92 | 0.068 |
| RWT*** | 0.42±0.04 | 0.41±0.03 | 0.011 |

Abbreviation: IVS, interventricular septal thickness; LVPWT, left ventricular posterior wall thickness; LVEDD, left ventricular end diastolic diameter; LVESD, left ventricular end systolic diameter; LAD, left atrial diameter; TR, tricuspid regurgitation; LVEDV, left ventricular end diastolic volume; LVESV, left ventricular end systolic volume; LAV, left atrial volume; LVEF, left ventricular ejection fraction; LVMI, left ventricular mass index; LAVI, left atrial volume index; RWT, relative wall thickness

*LVMI is calculated as left ventricular mass (LVM) normalized to body surface area (calculated by formula of Stevenson). LVM (g) = 0.8 × 1.04 × [IVS + LVEDD + LVPWT)^3^ − (LVEDD)^3]^ + 0.6

**Left atrial volume in mL is estimated using a validated equation derived from the linear antero-posterior dimension (LAD, in cm), measured in long-axis parasternal view ^[1]^, and then is normalized for body surface area to obtain LAVI

*** RWT is calculated as twice the LVPWT divided by LVEDD

References:

[1] Canciello G, de Simone G, Izzo R, et al. Validation of left atrial volume estimation by left atrial diameter from the parasternal long-axis view. J Am Soc Echocardiogr. 2017;30(3):262–9.

Supplementary Table 2. Comparison of prognostic value between NT-proBNP and resting cardiac power/mass

| Outcomes | C-index  (95%CI) | *P* | Continuous NRI  (%, 95%CI) | *P* | IDI  (%, 95%CI) | *P* |
| --- | --- | --- | --- | --- | --- | --- |
| Composite endpoint |  |  |  |  |  |  |
| Model + log NT-proBNP | 0.739(0.722–0.756) |  | 1.0(Ref) |  | 1.0(Ref) |  |
| Model + log NT-proBNP + resting cardiac power/mass | 0.742(0.729–0.755) | 0.002 | 5.0(1.0–10.0) | 0.020 | 13.2(2.1–23.5) | 0.036 |
| All-cause mortality |  |  |  |  |  |  |
| Model + log NT-proBNP | 0.753(0.724–0.781) |  | 1.0(Ref) |  | 1.0(Ref) |  |
| Model + log NT-proBNP + resting cardiac power/mass | 0.759(0.731–0.788) | 0.006 | 12.1(5.9–25.1) | 0.003 | 8.6(1.6–19.3) | 0.040 |
| Cardiovascular mortality |  |  |  |  |  |  |
| Model + log NT-proBNP | 0.891(0.872–0.910) |  | 1.0(Ref) |  | 1.0(Ref) |  |
| Model + log NT-proBNP + resting cardiac power/mass | 0.902(0.873–0.931) | 0.011 | 27.6(4.0–53.9) | 0.047 | 3.1(0.3–6.2) | 0.020 |
| HF hospitalization |  |  |  |  |  |  |
| Model + log NT-proBNP | 0.747(0.721–0.773) |  | 1.0(Ref) |  | 1.0(Ref) |  |
| Model + log NT-proBNP + resting cardiac power/mass | 0.749(0.723–0.775) | 0.002 | 17.3(1.9–29.1) | 0.035 | 5.3(1.2–9.4) | 0.013 |

Model is adjusted for age, gender, body mass index, New York Heart Association class, left ventricular ejection fraction, comorbidity score, estimated glomerular filtration rate, angiotensin-converting enzyme inhibitors/angiotensin receptor antagonist, beta blocker and aldosterone antagonist. CI, confidence interval; NRI, net re-classification improvement; IDI, integrated discrimination improvement; NT-proBNP, N-terminal pro-brain natriuretic peptide
